# Onion-derived activated carbons with enhanced surface area for improved hydrogen storage and electrochemical energy application†

**DOI:** 10.1039/d0ra04556j

**Published:** 2020-07-20

**Authors:** Nicholas M. Musyoka, Bridget K. Mutuma, Ncholu Manyala

**Affiliations:** Centre for Nanostructures and Advanced Materials (CeNAM), Chemicals Cluster, Council for Scientific and Industrial Research (CSIR) Meiring Naude Road Pretoria 0001 South Africa nmusyoka@csir.co.za +27-12-841-4806; Department of Physics, Institute of Applied Materials, SARCHI Chair in Carbon Technology and Materials, University of Pretoria South Africa bridgetmutuma@gmail.com Ncholu.manyala@up.ac.za

## Abstract

High surface area activated carbons (ACs) were prepared from a hydrochar derived from waste onion peels. The resulting ACs had a unique graphene-like nanosheet morphology. The presence of N (0.7%) and O content (8.1%) in the OPAC-800 °C was indicative of *in situ* incorporation of nitrogen groups from the onion peels. The specific surface area and pore volume of the best OPAC sample was found to be 3150 m^2^ g^−1^ and 1.64 cm^3^ g^−1^, respectively. The hydrogen uptake of all the OPAC samples was established to be above 3 wt% (at 77 K and 1 bar) with the highest being 3.67 wt% (800 °C). Additionally, the OPAC-800 °C achieved a specific capacitance of 169 F g^−1^ at a specific current of 0.5 A g^−1^ and retained a capacitance of 149 F g^−1^ at 5 A g^−1^ in a three electrode system using 3 M KNO_3_. A symmetric supercapacitor based on the OPAC-800 °C//OPAC-800 °C electrode provided a capacitance of 158 F g^−1^ at 0.5 A g^−1^. The maximum specific energy and power was found to be 14 W h kg^−1^ and 400 W kg^−1^, respectively. Moreover, the device exhibited a high coulombic efficiency of 99.85% at 5 A g^−1^ after 10 000 cycles. The results suggested that the high surface area graphene-like carbon nanostructures are excellent materials for enhanced hydrogen storage and supercapacitor applications.

## Abstract 2

Graphene-like activated carbons (ACs), with excellent properties for enhanced hydrogen storage and supercapacitor applications, were prepared from waste onion peels.

## Graphical abstract

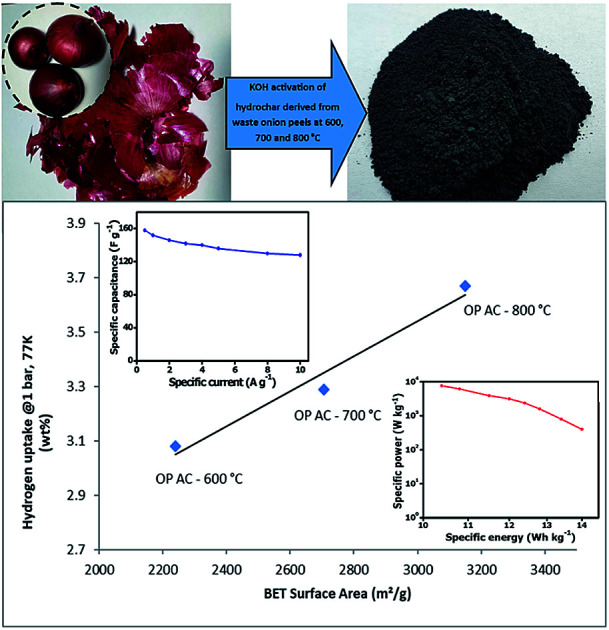

## 1. Introduction

Carbon-based porous materials have widely been used for hydrogen storage as well as in electrochemical energy storage applications. Even though these materials can be derived from many different feedstocks,^1,2^ the use of biomass is often preferred because it is easily available, low cost and also renewable.^2–4^ Examples of biomass that have been used to generate activated carbons (ACs) are such as tree bark,^5^ banana peel,^6^ algae,^2^ olive stones,^7^ among many others. The type of carbonaceous feedstock and the activation process play critical roles in determining the properties of the resulting ACs.^3,8^ For example, a recent study based on cellulose acetate-derived ACs with oxygen functional groups achieved a record-breaking hydrogen uptake value *i.e.* gravimetric capacity of 8.9 wt% at −196 °C and 30 bar.^9^ On the other hand, N-doped ACs have also received great interest in electrochemical energy storage applications since N presence leads to enhancement of the specific capacitance.^10,11^ The carbonization of N-containing biomass is also found to be cheaper and presents a straightforward approach for obtaining N-doped carbons devoid of using nitrogen-containing chemical precursors.

Owing to the need to identify a carbon precursor with high concentrations of oxygen and nitrogen functional groups, waste onion peels were considered as an attractive feedstock. In this case, onions (*Allium cepa* L.) are known to contain high oxygen functionalities due to the flavour precursors such as (+)-*S*-alk(en)yl cysteine sulphoxides (CSOs), *trans-S*-1-propenyl cysteine sulphoxide (PeCSO) and *S*-propyl cysteine sulphoxide (PCSO).^12^ The lachrymatory effect of onions is caused by presence of volatile propanthial *S*-oxide.^13^ A study by Venkateswarlu *et al.*^14^ confirmed that oxygen richness of carbonised onion peel aided As(iii) adsorption from contaminated water. On the other hand, the presence of N and S functionalities in ACs have also been reported to enhance hydrogen storage.^15–17^ Even though there are some studies that have reported the use of onion to derive ACs, these studies either used discarded green onion leaves^18^ or used un-optimised activation procedures^19^ which masked their true potential. Concerning the use of green onion leaves *versus* onion bulb scales, a study conducted by Lancaster *et al.*^20^ reported that the flavour precursors moves from the leaf blades towards the bulb scales which means that the concentration of oxygen-rich functionalities are often higher in onion bulb scales than in the leaves.

The abundance of onion skin waste, as a valorisable resource, can be evidenced by the availability of more than 500 000 metric tons which is discarded every year within the European Union.^21^ The utilisation of the discarded outer papery protective layers of onions in the production of ACs not only has the advantage of alleviating their disposal challenge but could lead to high properties of resulting ACs for enhanced hydrogen storage capacities. The nitrogen in the ACs would also enable better electrochemical capacitance owing to the increase in surface wettability and carbon hydrophilicity in aqueous electrolytes.

Herein, we report on the utilization of onion peels as the carbonaceous feedstock and KOH as the activating agent to produce nanosheet-like porous carbons. The effect of activation temperature on the structural and textural properties of the onion peel derived activated carbons (OPAC) was investigated. The obtained ACs exhibited a high specific surface area (2241–3150 m^2^ g^−1^) and an attractive pore volume (0.94–1.64 cm^3^ g^−1^). The OPAC material obtained at 800 °C (OPAC-800 °C) had the highest hydrogen uptake capacity of 3.67 wt% at 1 bar and 77 K. When used as the electrode material for supercapacitors in 3 M KNO_3_, the OPAC-800 °C achieved a specific capacitance of 169 F g^−1^ at 0.5 A g^−1^ in a three-electrode system. A fabricated symmetric device using the OPAC-800 °C displayed a capacitance of 158 F g^−1^ with corresponding specific energy and power of 14 W h kg^−1^ and 400 W kg^−1^ at 0.5 A g^−1^.

## 2. Experimental

### 2.1 Starting materials

Potassium hydroxide, KOH (85%, Merck), argon, Ar (99.99%, Afrox) and hydrochloric acid, HCl (37%, Merck) were used during the preparation of the onion derived ACs.

### 2.2 Preparation of the onion hydrochar

In this work, red onion skin wastes (peels) were collected, washed to remove excess dirt and then dried at 60 °C for 12 h. Thereafter, the onion-based hydrochar was obtained *via* hydrothermal carbonization which involved the heating of an aqueous mixture of the onion waste (300 g L^−1^) in an autoclave at 200 °C for 4 h. The resulting hydrochar was recovered by filtration, washed several times using deionised water and thereafter dried at 90 °C for 12 h.

### 2.3 Activation of the onion hydrochar

Pre-carbonization of the onion hydrochar was performed at 500 °C for 1 h under argon atmosphere with a ramp rate of 5 °C min^−1^. The pre-carbonized hydrochar was then mixed thoroughly with KOH at a mass ratio of 1 : 4 (hydrochar : KOH) and a few drops of water added to form a slurry which was then allowed to dry at 70 °C for 12 h. The mixture was then heated up to a pre-activation temperature of 400 °C for 30 min and then to an activation temperature of 600 °C for 1 h in a chemical vapour deposition reactor. The product was mixed with 10 wt% HCl for 24 h followed by washing with deionised water to a neutral pH and thereafter dried in an oven at 100 °C for 12 h to give the AC denoted as OPACs-600 °C. A similar procedure was used at activation temperatures of 700 °C and 800 °C to produce OPACs-700 °C and OPACs-800 °C, respectively.

### 2.4 Characterization

#### 2.4.1 General characterization and hydrogen storage testing

The ACs morphology was determined using a Zeiss Ultra Plus 55 field emission scanning electron microscope (FE-SEM) and a JEOL JEM-2100F transmission electron microscope operated at working voltage of 2 kV and 200 kV, respectively. The degree of graphitization of the ACs was investigated using a T64000 micro-Raman spectrometer (532 nm laser excitation wavelength and power of 5 mW). A PANalytical X'Pert Pro powder diffractometer was used for X-ray diffraction analysis. A Mettler, Toledo, TGA/SDTA 851e instrument was used for thermogravimetric analysis (TGA) which was conducted upto ∼1000 °C at a ramping rate of 10 °C min^−1^ under a mixture of air (60 mL min^−1^) and N_2_ (40 mL min^−1^). Micrometrics ASAP 2020 HD analyser was used for N_2_ and H_2_ sorption measurements at liquid N_2_ temperature (77 K) and up to 1 bar. The samples were firstly degassed for 8 h at 200 °C, under vacuum conductions (down to 10^−7^ bar), to remove any traces of adsorbed water and/or other physisorbed gases. X-ray photoelectron spectroscopy (XPS) analysis was conducted using a Physical Electronics Quantum 2000 and the data analysed using the CasaXPS software.

#### 2.4.2 Electrochemical characterization of the electrode materials

The onion-derived activated carbon electrode material, acetylene carbon black and polyvinylidene fluoride (PVDF) were mixed following a mass ratio of 80 : 10 : 10 and made into a slurry by adding a few drops of *N*-methyl-2-pyrrolidone (NMP). The resulting mixture was then coated onto a 1 cm^2^ nickel foam and dried under vacuum at 80 °C for 6 h. Electrochemical measurements were conducted using the Bio-Logic VMP300 potentiostat (Knoxville TN 37930, USA). Varying scan rates were employed during the collection of cyclic voltammetry (CV) data. Also, different specific current values were used for galvanostatic charge–discharge (GCD) measurements. A frequency range of 10 mHz to 100 kHz was used during the electrochemical impedance spectroscopy (EIS) measurements. A three-electrode conformation that consisted of the as-prepared OPAC material (as working electrode), glassy carbon counter electrode and Ag/AgCl reference electrode was assembled for electrochemical measurements in a 3 M KNO_3_ electrolyte solution. A coin cell configuration employing a Whatman separator and 3 M KNO_3_ was used for a symmetric device. The discharge curve of the GCD plot was used to calculate the specific capacitance for a half cell using eqn (1) whereas eqn (2) was used for the symmetric device. The specific energy and specific power of the device was determined using eqn (3) and (4).1
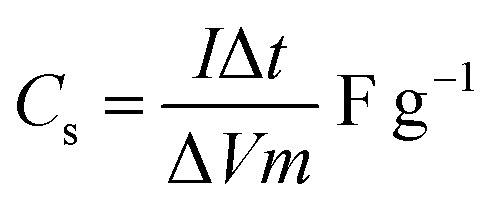
2
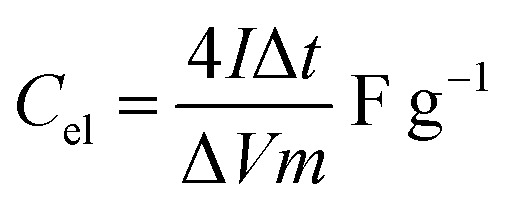
3
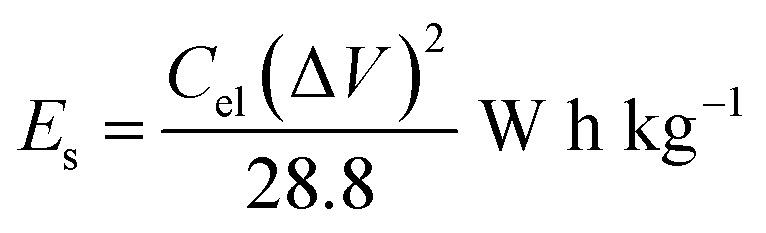
4
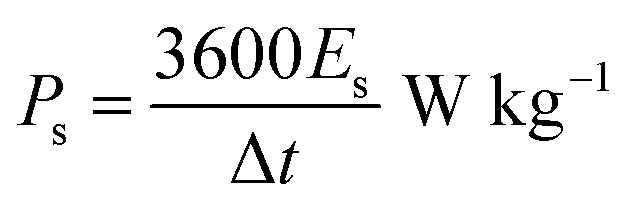
where *m* depicts the total mass of the electrode material, *I* is the current applied, Δ*t* being the discharge duration, *C*_el_ is the specific capacitance of a single electrode, and Δ*V* refers to the device voltage window.

## 3. Results and discussion

### 3.1 Morphological and structural analysis

**Fig. 1:**
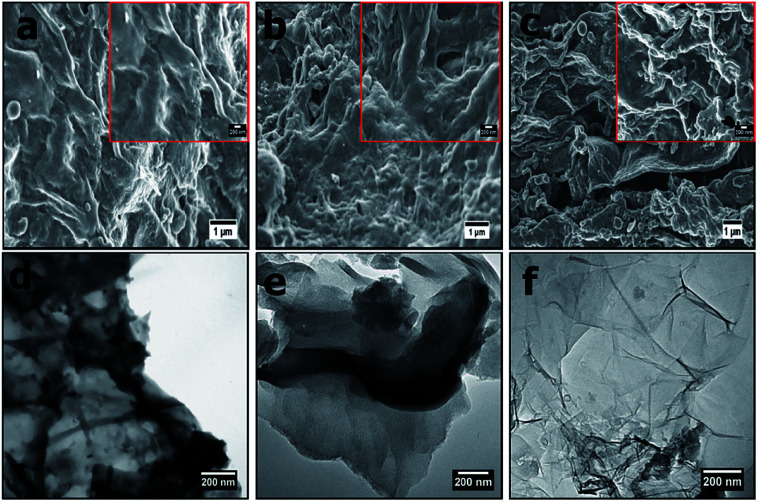
SEM images of (a) OPAC-600 °C, (b) OPAC-700 °C, (c) OPAC-800 °C taken at low magnification (insets shows the images at high magnification) and (d–f) their respective TEM images.

Fig. 1 presents morphological characteristics (SEM images) of the OPACs at high and low magnifications. The OPACs-600 °C and OPACs-700 °C displayed wrinkled carbon morphology (Fig. 1a and b). In contrast, the OPACs-800 °C exhibited a nanosheet-like morphology (Fig. 1c). The formation of the sheet-like structures at higher temperature could due to the exfoliation of staked onion cells after the intercalation of K. TEM images of the OPAC samples generated at 600 °C and 700 °C also shows the presence of overlapped wrinkled carbon nanostructures (Fig. 1d and e) whereas the OPAC-800 °C displayed transparent sheets with thinner walls (Fig. 1f). The observed crumpled nanosheet-like morphology of the OPAC samples can be correlated to the onion peels sheet-like structures. This observation reinforces the postulation that the structure of the raw material as well as the activation conditions plays an important role in determining the morphology of the resulting AC materials. In a different study, Venkateswarlu and his coworkers^14^ reported the generation of graphene-oxide like 2D carbon sheets after the pyrolysis of waste onion sheaths at 700 °C for 2 h in N_2_. Since the peels were obtained from mature onions, Turnbull *et al.*^22^ had earlier reported that the bulb cells of mature onions are often larger and thin walled. Therefore, we can postulate that upon the pre-carbonization of the onion hydrochar, the carbon wall tends to expand and this process can be accelerated by the expulsion of carbon dioxide during the activation step to lead to the creation of the observed nano-sheets that resembles graphene sheets.

**Fig. 2:**
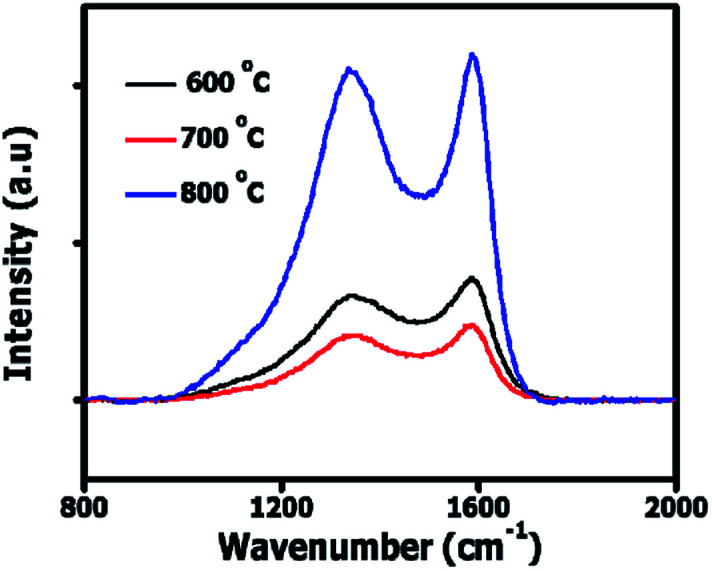
The Raman spectra of the onion-derived ACs.

Fig. 2 shows the Raman spectra of OPACs with D peaks observed at 1340–1344 cm^−1^ characteristic of the breathing mode of sp^2^ carbon atoms as well as the presence of sp^3^ hybridized amorphous carbon domains within the AC structure.^23,24^ A G peak attributed to the Raman active E_2g_ in-plane vibration mode of sp^2^ atoms in graphite was observed at 1586 cm^−1^.^25^

The upshift in the D band position as well as the observed increase in *I*_D_/*I*_G_ ratios relative to the activation temperature can be ascribed to the creation of more defects within the carbon matrix (Table 1). Besides, the Raman data shows that at 800 °C more amorphous domains were present in the ACs resulting in the high *I*_D_/*I*_G_ ratio displayed. Fig. S1† illustrates the XRD patterns of the OPAC samples. The presence of a broad hump coupled with the absence of a sharp peak depict the amorphous nature of sample. A weak broad diffraction peak at 2*θ* ∼ 43° was observed in the OPAC 600 and 700 °C samples, characteristic of (100) diffraction plane of graphite structure.^26^ However, for the OPAC 800 °C sample, this peak is barely visible and implies that graphitization decreased with the increase in the activation temperature. This observation correlates well with the Raman analysis presented in Table 1. Besides, in all the samples, the absence of the (002) diffraction plane of graphite peak at 2*θ* ∼ 24° was noted. This could suggest that a possible rearrangement of the structure and the creation of layer structure defects occurred due to K intercalation into the carbon structure.^27,28^ Fig. S2† present the TGA plots of the OPAC samples. The decomposition of the OPAC 600 °C started slightly below 400 °C whereas that of the OPAC 700 °C and 800 °C were at relatively higher temperature values.

**Table 1:** Comparative D and G band positions together with *I*_D_/*I*_G_ ratio of the onion-derived ACs

Material	D band position (cm^−1^)	G band position (cm^−1^)	*I*_D_/*I*_G_ ratio
OPAC-600 °C	1340	1586	0.86
OPAC-700 °C	1342	1586	0.87
OPAC-800 °C	1344	1586	0.96

### 3.2 Textural analysis

**Fig. 3:**
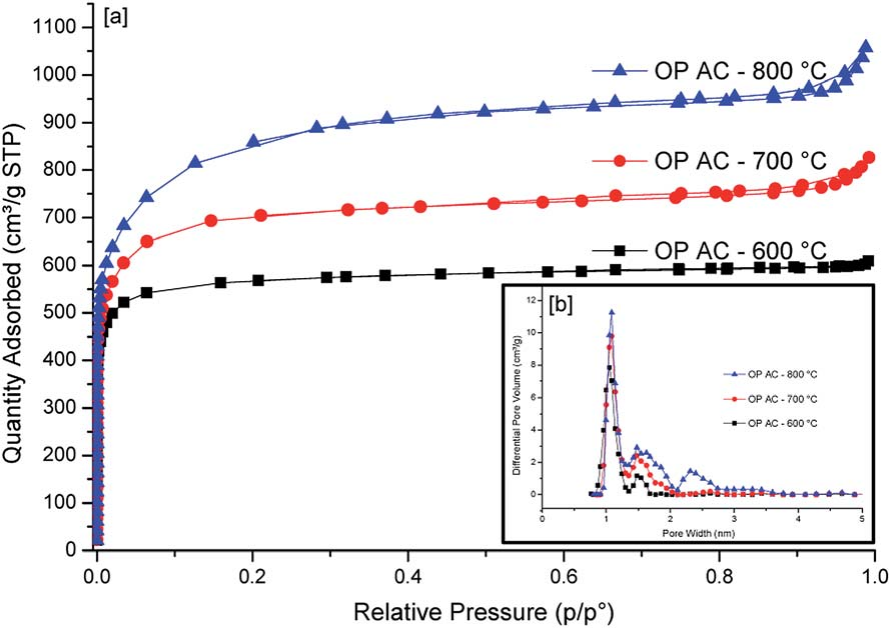
N_2_ sorption isotherms (a) together with pore side distribution curves (b) of OPACs produced at 600, 700 and 800 °C.

The N_2_ sorption isotherms of the three ACs generated from the three temperature variations are presented in Fig. 3. The sample generated at 600 °C exhibited a type I isotherm whereas those produced at 700 °C and 800 °C displayed both type I and IV isotherms. Type I isotherm is indicative of a microporous material having pores that are less than 2 nm while type IV isotherms depicts the presence of both micropores and mesopores.^29^ The sharp gas uptake in all the samples at low partial pressure region is a confirmation that most of the exposed surface resides mostly inside the micropores. This observation is further confirmed by the pore size distribution curves presented in Fig. 3b (inset). In this case, the samples produced at 600 °C and 700 °C portrayed a bimodal pore size distribution with the main pores having dimensions which are less than 2 nm (*i.e.* 1.05 and 1.08 nm, respectively). However, OPAC 800 °C sample exhibited a trimodal pore size distribution having pores positioned at 1.08, 1.48 and 2.31 nm. The presence of pores above 2 nm is an indication of the presence of mesopores which is further corroborated by the occurrence of the slight hysteresis loop in the isotherm.

The surface areas of the obtained ACs are presented in Table 2. The highest values were obtained for the sample generated at 800 °C (3150 m^2^ g^−1^) whereas the samples from 600 °C and 700 °C activation temperatures had 2706 m^2^ g^−1^ and 2241 m^2^ g^−1^, respectively. The temperature-dependent increase in the surface area can be attributed to the creation of more pores at higher temperatures. Typically, the KOH reacts with the carbon during the activation process to give K_2_CO_3_ and metallic K which intercalates into the carbon matrix.^30^ The K_2_CO_3_ then reacts with carbon resulting in a partial etching of carbon atoms, emission of CO gas and the intercalation of more K thus, creating additional pores on the carbon surface at higher activation temperatures (700 °C and 800 °C). Finally, the removal of the intercalated potassium by the washing of the final product with HCL increases the porosity of the carbon nanosheet. The unprecedented high surface areas are exceptional for biomass-derived carbons considering that the activating agent used was the conventional KOH without the inclusion of other special activation procedures. When compactivation strategy was applied by Mokaya's group,^31^ the compaction approach lead to ACs with 25% higher specific surface area. It is believed that the unexpected high specific surface areas obtained in our case could be due to the nature of the obtained morphological features that had sheet-like surface structures. Almost similar morphological features were obtained when activation of glucose was done using potassium oxalate.^3^ However, the specific surface areas were found to be relatively lower (1270–1690 m^2^ g^−1^) compared to our current study except in the case when a mixture of melamine and potassium oxalate was utilised. From Table 2, it was noted that the proportion of micropore area relative to the total surface area for all samples was over 97% with the OPAC-600 °C sample having the greatest proportion at 99.06%. On the higher hand, the trend observed for the temperature-dependent surface area was also noted for the pore volumes with the highest being for the OPAC-800 °C sample. It was also noted that the micropore volume represented ≥84% of the overall pore volume for the OPAC 700 and 800 °C activated samples with the highest being the low temperature activated carbon (600 °C) which was at ≥94%.

**Table 2:** Textural properties of OPACs produced at 600, 700 and 800 °C

Sample	BET surface area (m^2^ g^−1^)	Micropore area (m^2^ g^−1^)	Total pore vol. (cm^3^ g^−1^)	Micropore vol. (cm^3^ g^−1^)	H_2_ uptake (wt%)
OPAC-800 °C	3150	3080	1.64	1.39	3.67
OPAC-700 °C	2706	2651	1.28	1.10	3.29
OPAC-600 °C	2241	2220	0.94	0.89	3.08

### 3.3 Elemental composition by XPS analysis

**Fig. 4:**
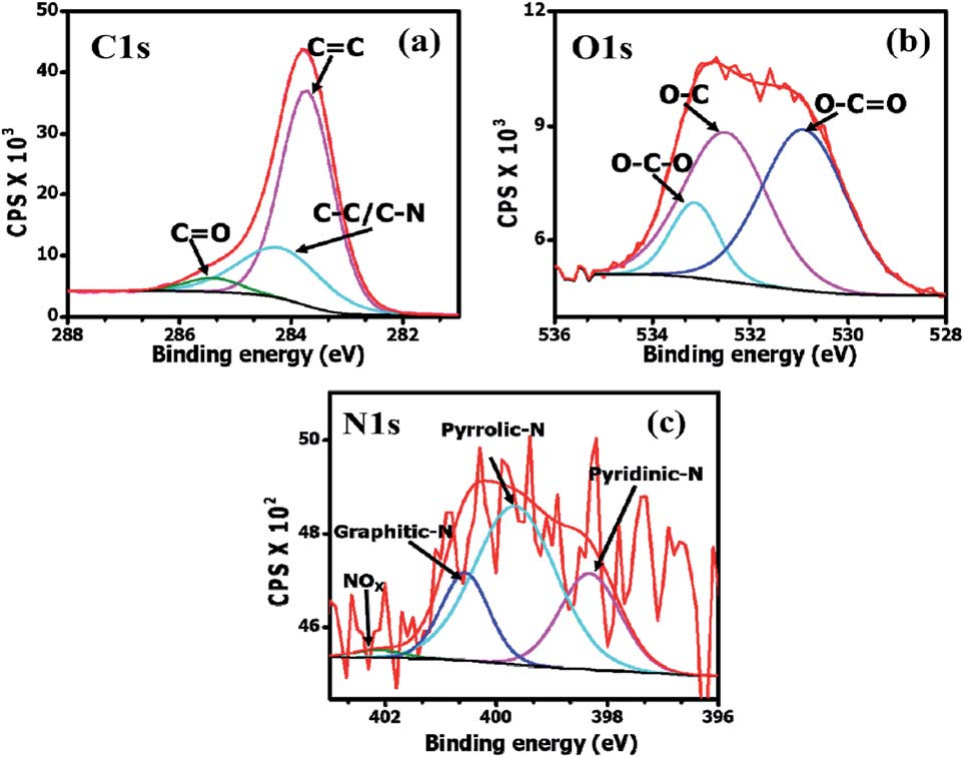
The deconvoluted (a) C 1s, (b) O 1s and (c) N 1s spectra for the OPAC-800 °C.

The elemental composition of the OPAC-800 °C sample was determined using X-ray photoelectron spectroscopy. The integral areas of the deconvoluted C 1s, O 1s and N 1s peaks were used to calculate the atomic percentages present in the material. After pre-carbonization and KOH activation at 800 °C, the oxygen, carbon and nitrogen contents were 8.1 at%, 91.2 at% and 0.7 at%, respectively. The low nitrogen content can be ascribed to the loss of nitrogenous products at the high activation temperature.^32^Fig. 4a shows the deconvoluted C 1s peaks namely; sp^2^ C=C (283.7; 69.7%), sp^3^ C–C/C–N (284.3 eV; 25.8%) and C=O bonds (285.5; 4.5%), respectively.^33,34^ The higher percentage of the sp^2^-hybridized carbons as compared to the sp^3^ carbon can be ascribed to the restoration of the graphitic carbons within the AC matrix at the high activation temperature (800 °C). From Fig. 4b, the deconvoluted O 1s spectrum presented peaks centered at 530.9 eV (O–C=O), 532 eV (O–C) and 533.5 eV (O–C–O). Fig. 4c displays the deconvoluted N 1s for the OPAC-800 °C sample with spectrum peaks centered at 398.3 (24.8%), 399.7 (55.7%), 400.6 (17.9%) and 402.1 (1.6%) which corresponds to the pyridinic, pyrrolic, graphitic N atoms and the oxidized pyridinic-N, respectively.^35,36^ The presence of pyridinic-N and pyrrolic-N is known to readily promote carbon surface wettability, which consequently enhances the electrochemical capacitive properties.^35,37,38^

### 3.4 Hydrogen uptake

**Fig. 5:**
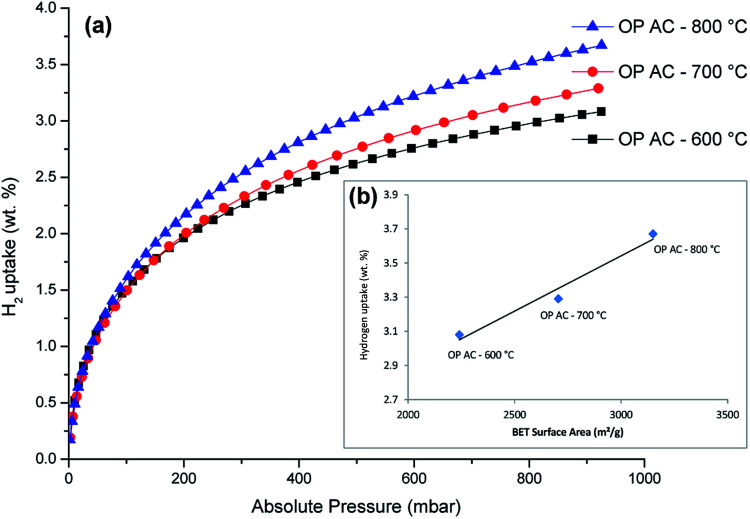
H_2_ sorption isotherms of OPACs produced at 600, 700 and 800 °C (a) and the respective relationship of H_2_ uptake with the surface areas (inset, (b)).

The hydrogen sorption isotherms are presented in Fig. 5. All the three samples had a H_2_ uptake that was above 3 wt% at 77 K and 1 bar (as also shown in Table 2). The lack of a hysteresis loop in the isotherms meant that the H_2_ uptake was reversible. Noteworthy, no saturation was attained at the analysis pressure which is an indication that higher H_2_ sorption capacities would be expected at higher pressure ranges. The impressive hydrogen uptake at 1 bar (3.08–3.67 wt%) was comparatively higher than values often reported for typical porous carbons which are usually within the range of 2–3 wt%.^9^ Most importantly, the best hydrogen uptake value of 3.67 wt% up to 1 bar at 77 K obtained using the onion peels derived activated carbons is superior to most reported data for other related materials^28,39–44^, as listed in Table 3. The abundance of narrow pores (nano and micro levels) in a porous material has been reported to make the biggest contribution to H_2_ adsorption capacity^45–48^ and hence the observed enhancements of the performance of the activated carbon in this study.

**Table 3:** Comparative performance of various activated carbons materials for hydrogen storage and supercapacitor applications

Materials	Method	Specific surface area (m^2^ g^−1^)	Hydrogen storage	Electrolyte (2-electrode system)	Specific energy	Ref.
Hemp (*Cannabis sativa* L.) stem	Carbonization and KOH activation	2436	2.84 wt% at 1 bar, 77 K	—	—	39
Resorcinol–formaldehyde	KOH activation	915	2.7 wt% at 20 bar/77 K	—	—	40
Corncob	Carbonization and KOH activation	3530	2.66 wt% at 1 bar, 77 K	—	—	41
Rice husks	KOH activation	2682	2.85 wt% at 1 bar/77 K	—	—	28
Chitosan	Carbonization and KOH activation	2481	2.95 wt% at 0.1 MPa/77 K	—	—	65
Empty fruit bunch	KOH and CO_2_ activation	687	2.14 wt% at 20 bar/77 K	—	—	42
Cornstalk	Fe(CN)_6_ complex and carbonization	540	—	6 M KOH	9.4 W h kg^−1^ at 0.5 A g^−1^	60
Pistachio nutshells	KOH activation and carbonization	1069	—	6 M KOH	10 W h kg^−1^ at 0.2 A g^−1^	61
Shiitake mushroom	H_3_PO_4_ and KOH activation and carbonization	2988	—	6 M KOH	8.2 W h kg^−1^ at 3 A g^−1^	62
Camelia petals	(NH_4_)_2_S_2_O_8_ treatment and carbonization	1122	—	6 M KOH	7.8 W h kg^−1^ at 0.5 A g^−1^	63
Cork (*Quercus suber*)	Acid pre-treatment, KHCO_3_ activation and carbonization	1057	—	3 M KNO_3_	14 W h kg^−1^ at 0.5 A g^−1^	64
Kadamba (*Neolamarckia cadamba*)	Carbonization and KOH activation	3192	2.71 wt% at 1 bar/77 K	6 M KOH	11.1 W h kg^−1^ at 0.2 A g^−1^	43
Sword bean shells	Carbonization and KOH activation	2838	2.63 wt% at 1 bar/77 K	6 M KOH	19.5 W h kg^−1^ at 10 A g^−1^	44
**Onion peels**	**Carbonization and KOH activation**	**3100**	**3.67 wt% at 1 bar/77 K**	**3 M KNO**_**3**_	**14 W h kg** ^**−1**^ **at 0.5 A g** ^**−1**^	**This work**

As shown in Fig. 5b, a linear correlation of the specific surface area with the hydrogen uptake capacity for the OPAC samples was also observed in this study. This observation is in agreement with Chahine's rule which highlights the generally accepted direct proportionality trend of increasing H_2_ gravimetric capacity with increasing surface area commonly observed in nanoporous materials.^49^ It is also thought that the presence of oxygen functionality (as confirmed by XPS) could have also played a role in the observed high H_2_ adsorption capacity. In this regard, an earlier study by Blankenship II *et al.*^9^ had reported that a combined effect of high surface area, oxygen-rich functionality and high microporosity contributed to the enhancement of gravimetric hydrogen storage capacity. The researchers further alluded that the oxygen beneficial effect is more pronounced at low pressure (∼1 bar) region owing to the fact that the interaction between the hydrogen and the material surface is more intimate when compared to the higher pressure ranges where hydrogen uptake is most likely to happen by space filling mechanisms. Even though high H_2_ uptake values are often reported for analyses conducted at cryogenic temperatures, doping the sorbent materials with heteroatoms or inclusion of metal particles (such as Pt) can enhance hydrogen storage capacities at room temperature.^50^ These strategies could be applied to the OPACs materials obtained in this study.

### 3.5 Electrochemical performance

**Fig. 6:**
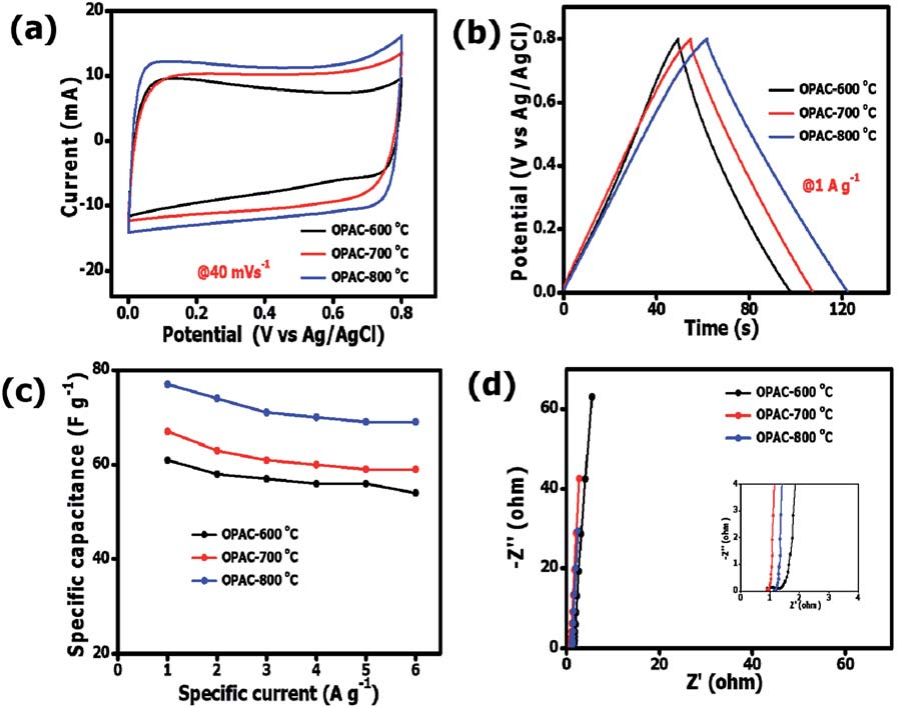
(a) CV curves, (b) GCD curves, (c) plots of specific capacitance as a function of specific current and (d) Nyquist impedance plots of all the OPAC materials in a 3 M KNO_3_ electrolyte solution.

The evaluation of electrochemical performance of the as-synthesized OPAC electrode materials was based on a three-electrode configuration using a 3 M KNO_3_ electrolyte solution. Fig. 6a presents the cyclic voltammograms of all the electrode materials taken at 40 mV s^−1^. The operating cell potential of 0.8 V was chosen as no overpotential peak as observed in Fig. S3a† for both negative and positive potential. For the 3 samples, a quasi-rectangular CV curve was observed indicating a typical electrical double layer capacitor (EDLC) property.^51^ However, the CV curve for the OPAC-600 °C was slightly distorted suggesting that the material has more oxygenated functional groups.^52^Fig. 6b displays the quasi-symmetrical galvanostatic charge–discharge plots obtained at 1 A g^−1^ specific current in an operating cell potential of 0.0 to 0.8 V. The calculated specific capacitance values at 1 A g^−1^ were 61, 67 and 77 F g^−1^ for the OPAC-600 °C, OPAC-700 °C and OPAC-800 °C, respectively. It was found that the OPAC-800 °C gave the highest current response as well as the longest discharge time when compared to the others. From Fig. 6c, it was evident that there was a direct correlation of specific capacitance and activation temperature with the highest capacitance recorded for OPAC-800 °C sample. This observation can be attributed to the sample's improved pore volume and high specific surface area. The electrochemical responses of the as-prepared electrode materials were also examined in the negative potential range of −0.8 to 0.0 V in 3 M KNO_3_. Fig. S3b–d† shows the CV curves, GCD plots and specific capacitance *versus* specific current displaying a similar trend to what was observed in the positive potential range. From Fig. S3d,† the respective specific capacitance values for the OPAC-600 °C, OPAC-700 °C and OPAC-800 °C samples were found to be 120, 146 and 157 F g^−1^ when measured at a specific current value of 1 A g^−1^. The higher specific capacitance of the OPAC-800 °C can be ascribed to its higher surface area that provided better electrolyte ion accessibility to the pores within the carbon matrix. Good rate capability was observed for all the electrode materials.

The impedance properties of the OPAC materials at an operating frequency of 10 mHz to 100 kHz (Fig. 6d) was studies using electrochemical impedance spectroscopy (EIS) technique. The OPAC-800 °C displayed the shortest diffusion length indicating faster ion diffusion within the material. The OPAC-600 °C curve was found to deviate slightly from the vertical axis depicting a less capacitive behaviour as compared to its counterparts. The intersection of curves with the horizontal axis describes the equivalent series resistance (ESR) which is a sum total of the active materials intrinsic resistance, electrolyte resistance, and the contact resistance occurring between the electrode materials and the current collector.^53–55^ The ESR was measured to be 0.90, 0.91 and 1.14 Ω for OPAC-600 °C, OPAC-700 °C and OPAC-800 °C, respectively. The OPAC-600 °C depicted a large semicircle with a charge transfer resistance value of 1.02 Ω. In contrast, the OPAC-800 °C had the smallest semicircle corresponding to a charge transfer resistance value of 0.03 Ω and the shortest diffusion length, which are responsible for the improved electrochemical performance.

**Fig. 7:**
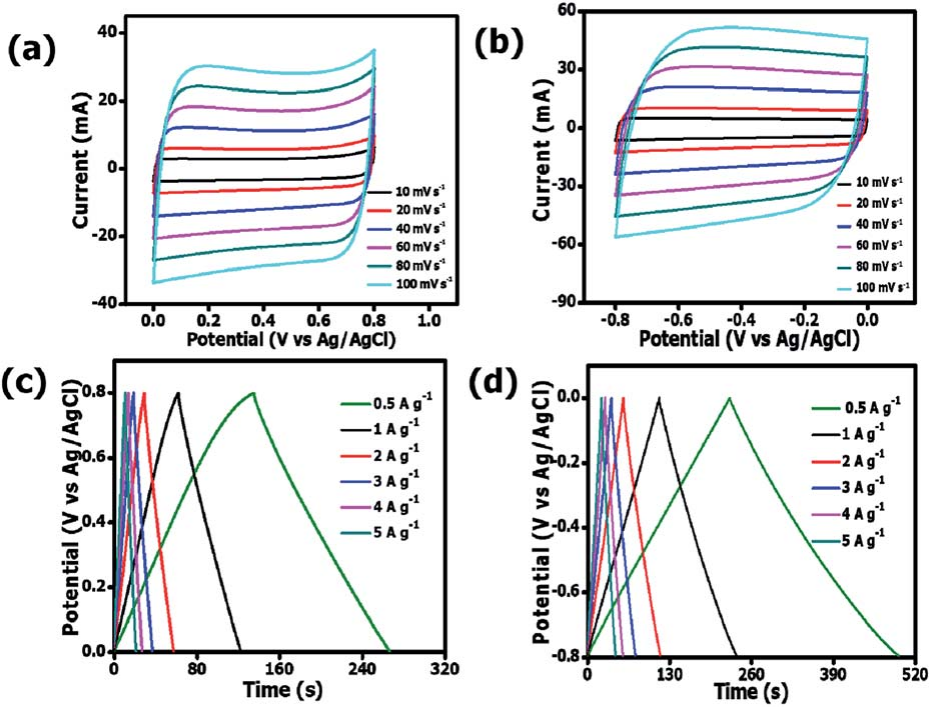
CV curves (a, b) and GCD plots (c, d) of the OPAC-800 °C at a potential range of −0.8 V to 0.8 V in 3 M KNO_3_ electrolyte.

Fig. 7a and b present CV curves of OPAC-800 °C sample that were obtained at various scan rates (10 to 100 mV s^−1^) both in the negative and positive operating potentials, ranging from −0.8 to 0 V and 0 to 0.8 V, respectively. The figures show that the materials could operate well even within a reversible potential range.

A quasi-rectangular shape had been maintained even at higher scan rates of 100 mV s^−1^ depicting the fast ion diffusion kinetics on the reversal of potential.^56^Fig. 7c and d show the GCD plots for the OPAC-800 °C tested at different specific current values of 0.5 A g^−1^ to 6 A g^−1^ in 3 M KNO_3_ electrolyte solution. The calculated specific capacitance values were 169, 157, 151, 150, 149 and 149 F g^−1^ at 0.5, 1, 2, 3, 4 and 5 A g^−1^ in the potential range of −0.8 to 0.0 V. This shows that a good rate capability of 88% was maintained at higher specific current of 5 A g^−1^. At positive potential ranges of 0.0 to 0.8 V, the specific capacitance values were 83, 77, 74, 71, 70 and 69 F g^−1^ at 0.5, 1, 2, 3, 4 and 5 A g^−1^, respectively. It is observed that the OPAC-800 °C showed good rate capability at the distinct specific currents. As a result of the significant stability of the OPAC-800 °C electrode material, an assembly of a symmetric supercapacitor was deemed worthwhile.

**Fig. 8:**
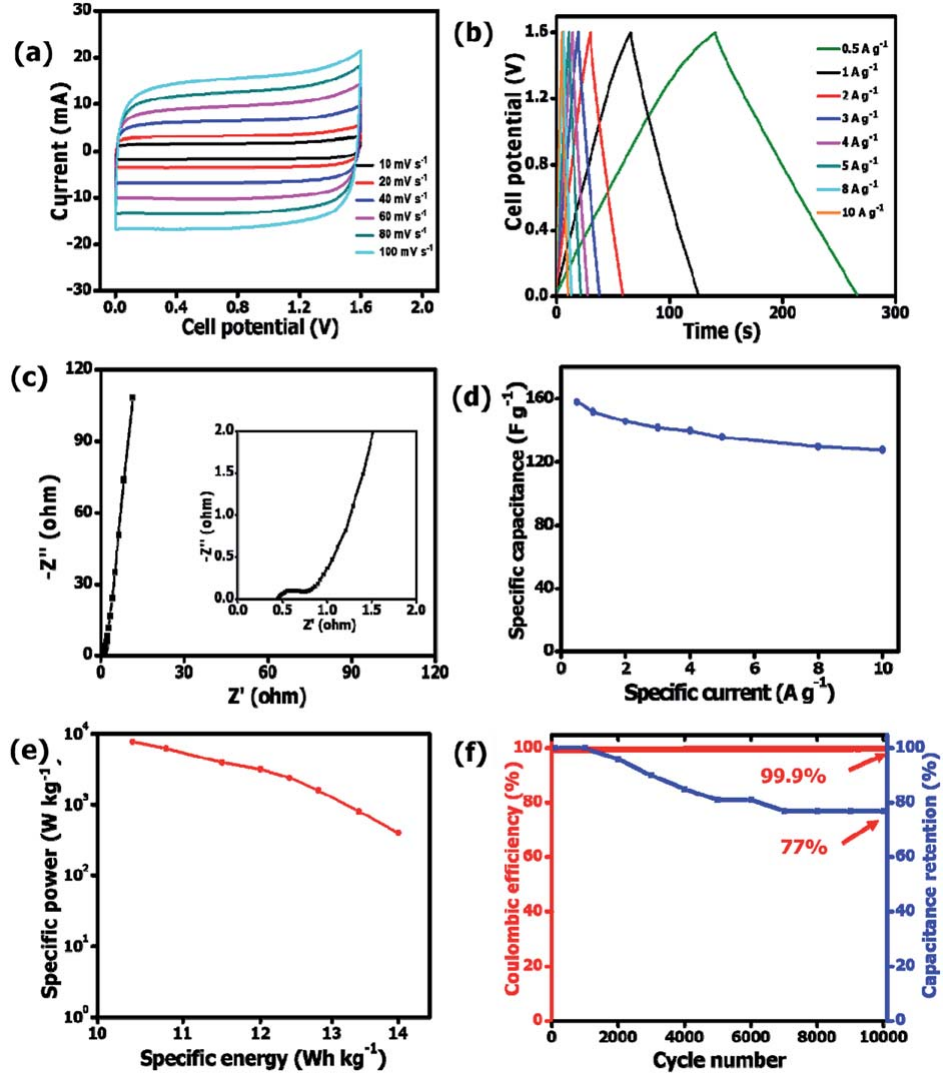
CV curves at distinct scan rates, (b) GCD plots at various specific currents, (c) specific capacitance determined at various specific currents, (d) Nyquist impedance plots, (e) Ragone plot and (f) cycle stability and associated capacitance retention for the OPAC 800 °C//OPAC 800 °C symmetric device.

A two-electrode setup in 3 M KNO_3_ electrolyte was used to investigate the electrochemical performance of the symmetric device because of the inherent potential that was exhibited from the half-cell analysis. Fig. 8a shows the CV curves of symmetric OPAC 800 °C//OPAC 800 °C device conducted at varying scan rates of between 10 and 100 mV s^−1^. The associated GCD profiles of the full device at various specific current are presented in Fig. 8b. The obtained GCD curves as well as the CV curves were found to be fairly symmetrical and rectangular, respectively, which is in accordance to the EDLC nature of activated carbon symmetric devices.^57^ The Nyquist plot for the symmetric device with an equivalent series resistance of 0.44 Ω and a charge transfer resistance of 0.31 Ω is presented in Fig. 8b. The ESR value was smaller compared to that reported for similar activated carbon.^58,59^Fig. 8d shows the specific capacitance of the symmetric SC that was plotted against the gravimetric specific current. The symmetric device was found to produce a maximum specific capacitance of 158 F g^−1^ at a specific current of 0.5 A g^−1^. Fig. 8e illustrates a Ragone plot for both specific energy and specific power of the symmetric device which are quite significant in the application of supercapacitors in real-life situations. At a specific current of 0.5 A g^−1^, specific energy of 14.0 W h kg^−1^ corresponding to specific power of 400 W kg^−1^ was achieved. While at a specific current of 1 A g^−1^, a specific energy of 13.4 W h kg^−1^ corresponding to a specific energy of 800 W kg^−1^ was obtained. At 10 A g^−1^, specific energy of 10.4 W h kg^−1^ and corresponding specific power of 7655 W kg^−1^ was maintained. The symmetric device exhibited a coulombic efficiency of 99.9% and conserved 77% of the initial capacitance after 10 000 charge and discharge cycles at a specific current of 5 A g^−1^ (Fig. 8f). This can be ascribed to the high specific surface area as well as the interconnected porous network of the OPAC-800 °C.

Table 3 shows that the OPAC 800 °C device presented not only good specific capacitance and excellent capacitance retention (99.9%) but also exhibited good specific energy, which was comparable to other related materials in literature.^43,44,60–64^ From Table 3, the high specific capacitance for the OPAC-800 °C electrode material obtained in this study is due to its high specific surface area, enhanced pore volume as well as the high *I*_D_/*I*_G_ ratios. These properties allows easy access of the electrolyte ions into the pores and enables better surface wettability. Moreover, the presence of trimodal pores in the nanosheet framework created an interconnected pore network for good energy storage. Finally, the oxygen and nitrogen functionalities play a crucial role in the influencing the hydrophilicity of the electrode surface which consequently impacts on its electrochemical performance. These results show the potential for the use of onion-peels and skins as a suitable biomass source in the generation of activated carbon nanosheets for application in hydrogen storage and electrochemical capacitors.

## 4. Conclusions

This study showed that activated carbons prepared from waste onion sheaths using KOH, as the activating reagent, exhibited exceptional properties that made them desirable for hydrogen storage and electrochemical applications. The resulting ACs were found to have a nano-sheet type of morphology and had a moderate concentration of nitrogen and oxygen functional groups. The highest specific surface area (3150 m^2^ g^−1^) and pore volume (1.64 cm^3^ g^−1^) was achieved when activation was conducted at 800 °C. Noteworthy, hydrogen sorption (at 77 K and 1 bar) for the 3 activated samples was above 3 wt% with the highest being 3.67 wt% when activation was conducted at 800 °C. These values were comparatively higher than those often reported for typical porous carbons which usually range from 2 to 3 wt%. Furthermore, the obtained ACs samples also exhibited commendable electrochemical performance, by having a specific capacitance of 158 F g^−1^ (at a specific current of 0.5 A g^−1^), specific energy of 14 W h kg^−1^, power of 400 W kg^−1^ and with a capacitance retention of 77% at 5 A g^−1^ even after 10 000 charge–discharge cycles. The results demonstrated that the onion derived ACs could be considered as promising candidates for hydrogen storage and electrochemical energy storage applications.

## Conflicts of interest

There are no conflicts of interest to declare.

## Supplementary Material

RA-010-D0RA04556J-s001

## References

[cit1] Heidarinejad Z., Dehghani M. H., Heidari M., Javedan G., Ali I., Sillanpää M. (2020). Environ. Chem. Lett..

[cit2] Zhu B., Liu B., Qu C., Zhang H., Guo W., Liang Z. (2018). J. Mater. Chem. A.

[cit3] Sevilla M., Al-Jumialy A. S. M., Fuertes A. B., Mokaya R. (2018). ACS Appl. Mater. Interfaces.

[cit4] Sangchoom W., Walsh D. A., Mokaya R. (2018). J. Mater. Chem. A.

[cit5] Momodu D., Madito M., Barzegar F., Bello A., Khaleed A., Olaniyan O., manyala N. (2017). J. Solid State Electrochem..

[cit6] Fasakin O., Dangbegnon J. K., Momodu D. Y., Madito M. J., Oyedotun K. O., Eleruja M. A., Manyala N. (2018). Electrochim. Acta.

[cit7] Saleem J., Shahid U. B., Hijab M., Mackey H., McKay G. (2019). Biomass Convers. Biorefin..

[cit8] Jain A., Balasubramanian R., Srinivasan M. P. (2016). Chem. Eng. J..

[cit9] Blankenship II T. S., Balahmar N., Mokaya R. (2017). Nat. Commun..

[cit10] Wang K., Zhao N., Lei S., Yan R., Tian X., Wang J. (2015). Electrochim. Acta.

[cit11] Chen X., Zhang J., Zhang B., Dong S., Guo X., Mu X. (2017). Sci. Rep..

[cit12] Jones M. G., Hughes J., Tregova A., Milne J., Tomsett A. B., Collin H. A. (2004). J. Exp. Bot..

[cit13] Brodnitz M. H., Pascale J. V. (1971). J. Agric. Food Chem..

[cit14] Venkateswarlu S., Lee D., Yoon M. (2016). ACS Appl. Mater. Interfaces.

[cit15] Xia Y., Walker G. S., Grant D. M., Mokaya R. (2009). J. Am. Chem. Soc..

[cit16] Xia Y., Zhu Y., Tang Y. (2012). Carbon.

[cit17] Zheng M., Zhang H., Xiao Y., Dong H., Liu Y., Xu R. (2013). Mater. Lett..

[cit18] Yu J., Gao L., Li X., Wu C., Gao L., Li C. (2016). N. Carbon Mater..

[cit19] Anitha A., Kalyani P. (2014). Int. J. Curr. Res..

[cit20] Lancaster J. E., McCallion B. J., Shaw M. L. (1986). Physiol. Plant..

[cit21] Choi I. S., Cho E. J., Moon J.-H., Bae H.-J. (2015). Food Chem..

[cit22] Turnbull A., Galpin I., Smith J., Collin H. (1981). New Phytol..

[cit23] Burke A. (2009). J. Power Sources.

[cit24] Simon P. and Gogotsi Y., Materials for electrochemical capacitors, in Nanosci. Technol., co-published with Macmillan Publishers Ltd, UK, 2009, pp. 320–329

[cit25] Zhang L. L., Zhao X. S. (2009). Chem. Soc. Rev..

[cit26] Rao C. N. R., Biswas K., Subrahmanyam K. S., Govindaraj A. (2009). J. Mater. Chem..

[cit27] Meng L.-Y., Park S.-J. (2010). J. Colloid Interface Sci..

[cit28] Heo Y.-J., Park S.-J. (2015). J. Ind. Eng. Chem..

[cit29] Sing K. S. W., Everett D. H., Haul R. A. W., Moscou L., PierottI R. A., Rouquerol J. (1985). Pure Appl. Chem..

[cit30] Wang J., Kaskel S. (2012). J. Mater. Chem. A.

[cit31] Balahmar N., Mitchell A. C., Mokaya R. (2015). Adv. Energy Mater..

[cit32] Hulicova D., Yamashita J., Soneda Y., Hatori H., Kodama M. (2005). Chem. Mater..

[cit33] Díaz J., Paolicelli G., Ferrer S., Comin F. (1996). Phys. Rev. B: Condens. Matter Mater. Phys..

[cit34] Mutuma B. K., Matsoso B. J., Ranganathan K., Keartland J. M., Wamwangi D., Coville N. J. (2017). RSC Adv..

[cit35] Hulicova-Jurcakova D., Seredych M., Lu G. Q., Bandosz T. J. (2009). Adv. Funct. Mater..

[cit36] Gao S., Chen Y., Fan H., Wei X., Hu C., Luo H. (2014). J. Mater. Chem. A.

[cit37] Lota G., Grzyb B., Machnikowska H., Machnikowski J., Frackowiak E. (2005). Chem. Phys. Lett..

[cit38] Kwon T., Nishihara H., Itoi H., Yang Q., Kyotani T. (2009). Langmuir.

[cit39] Yang R., Liu G., Li M., Zhang J., Hao X. (2012). Microporous Mesoporous Mater..

[cit40] Robertson C., Mokaya R. (2013). Microporous Mesoporous Mater..

[cit41] Zhang C., Geng Z., Cai M., Zhang J., Liu X., Xin H. (2013). Int. J. Hydrogen Energy.

[cit42] Arshad S. H., Ngadi N., Aziz A. A., Amin N. S., Jusoh M., Wong S. (2016). J. Energy Storage.

[cit43] Hu W., Huang J., Yu P., Zheng M., Xiao Y., Dong H. (2019). ACS Sustainable Chem. Eng..

[cit44] Chen T., Zhou Y., Luo L., Wu X., Li Z., Fan M. (2019). Electrochim. Acta.

[cit45] Nishihara H., Kyotani T. (2012). Adv. Mater..

[cit46] Yang Z., Xia Y., Mokaya R. (2007). J. Am. Chem. Soc..

[cit47] Thomas K. M. (2007). Catal. Today.

[cit48] Alam N., Mokaya R. (2010). Energy Environ. Sci..

[cit49] Broom D. P., Webb C. J., Fanourgakis G. S., Froudakis G. E., Trikalitis P. N., Hirscher M. (2019). Int. J. Hydrogen Energy.

[cit50] Juárez J. M., Costa M. B., Anunziata O. A. (2015). Int. J. Energy Res..

[cit51] Fic K., Lota G., Meller M., Frackowiak E. (2012). Energy Environ. Sci..

[cit52] Le Van K., Luong Thi T. T. (2014). Prog. Nat. Sci.: Mater. Int..

[cit53] Kötz R., Carlen M. (2000). Electrochim. Acta.

[cit54] Mei B.-A., Munteshari O., Lau J., Dunn B., Pilon L. (2018). J. Phys. Chem. C.

[cit55] Gamby J., Taberna P., Simon P., Fauvarque J. F., Chesneau M. (2001). J. Power Sources.

[cit56] Zhou J., Zhu T., Xing W., Li Z., Shen H., Zhuo S. (2015). Electrochim. Acta.

[cit57] Liu C. L., Dong W. S., Cao G. P., Song J. R., Liu L., Yang Y. S. (2007). J. Electroanal. Chem..

[cit58] Show Y., Imaizumi K. (2006). Diamond Relat. Mater..

[cit59] Momodu D., Bello A., Oyedotun K., Ochai-Ejeh F., Dangbegnon J., Madito M., Manyala N. (2017). RSC Adv..

[cit60] Wang L., Mu G., Tian C., Sun L., Zhou W., Yu P. (2013). ChemSusChem.

[cit61] Xu J., Gao Q., Zhang Y., Tan Y., Tian W., Zhu L. (2014). Sci. Rep..

[cit62] Cheng P., Gao S., Zang P., Yang X., Bai Y., Xu H. (2015). Carbon.

[cit63] Wei T., Wei X., Yang L., Xiao H., Gao Y., Li H. (2016). J. Power Sources.

[cit64] Ochai-Ejeh F. O., Momodu D. Y., Madito M. J., Khaleed A. A., Oyedotun K. O., Ray S. C., Manyala N. (2018). AIP Adv..

[cit65] Wróbel-Iwaniec I., Díez N., Gryglewicz G. (2015). Int. J. Hydrogen Energy.

