# Supplementary material for: Onion-derived activated carbons with enhanced surface area for improved hydrogen storage and electrochemical energy application
Source: RSC Adv. 2020 Jul 20;10(45):26928–36. doi: 10.1039/d0ra04556j (PMC9055538; doi:10.1039/d0ra04556j)
Supplement: RA-010-D0RA04556J-s001 [file RA-010-D0RA04556J-s001.pdf]

Electronic Supplementary Material (ESI) for RSC Advances.
This journal is © The Royal Society of Chemistry 2020

Supplementary Information

Onion-derived Activated Carbons with Enhanced Surface Area for Improved Hydrogen Storage and Electrochemical Energy Application

Nicholas M. Musyoka[1,], Bridget K. Mutuma[2*], Ncholu Manyala[2*]*

[1]HySA Infrastructure Centre of Competence, Chemicals Cluster, Council for Scientific and Industrial Research (CSIR), Meiring Naude Road, Pretoria, South Africa

[2]Department of Physics, Institute of Applied Materials, SARCHI Chair in Carbon Technology and Materials, University of Pretoria, South Africa

E-mail address: nmusyoka@csir.co.za, bridgetmutuma@gmail.com, ncholu.manyala@up.ac.za

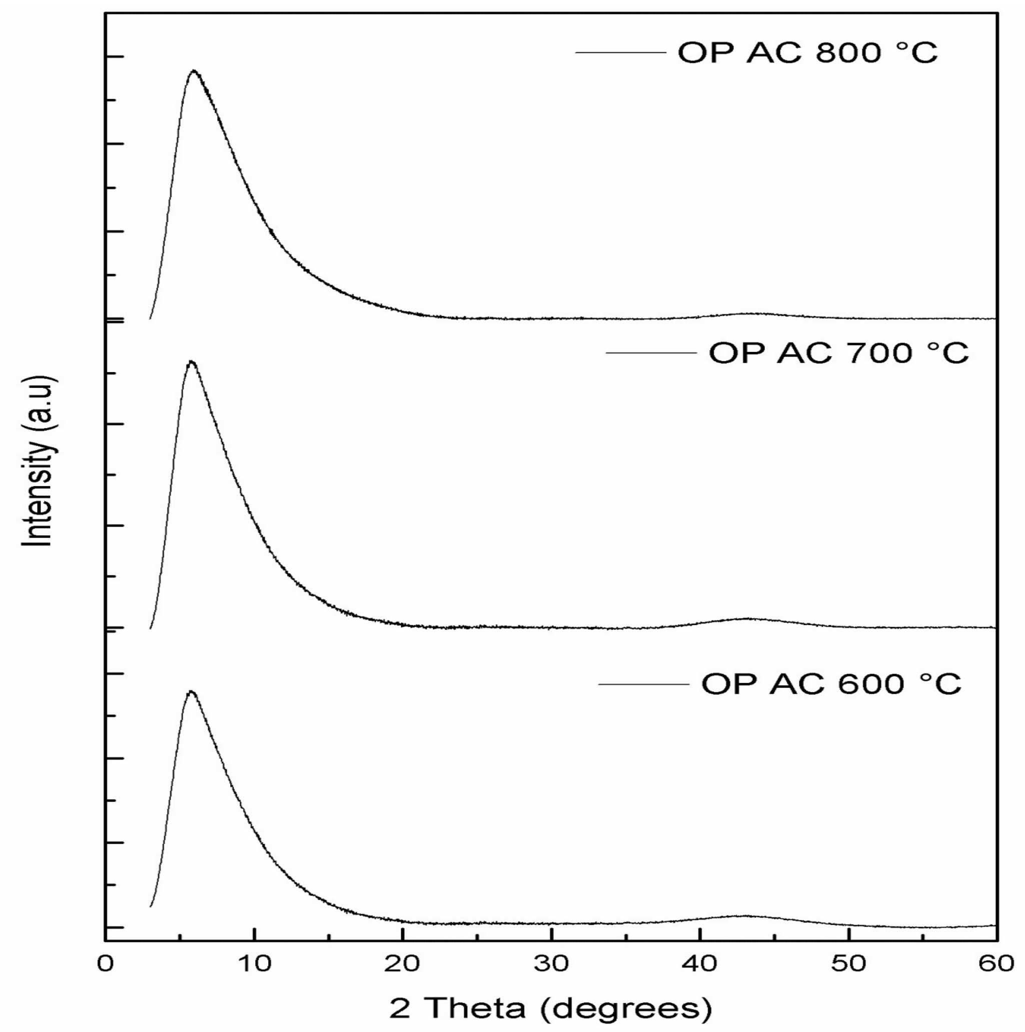

Figure S1: XRD patterns of OP ACs produced at 600, 700 and 800 °C.

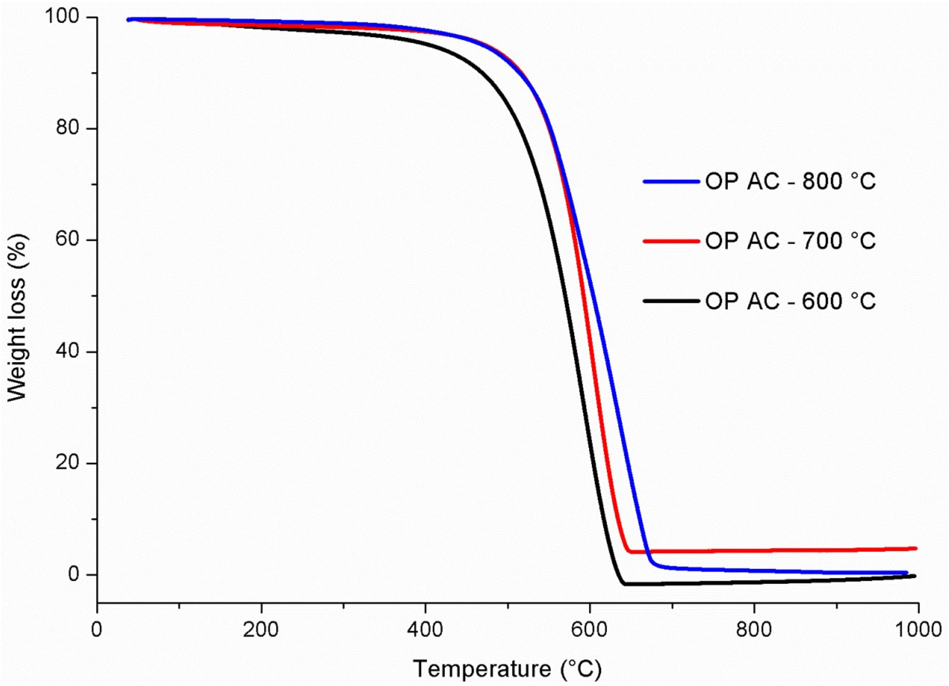

Figure S2: TGA plots of OP ACs produced at 600, 700 and 800 °C.

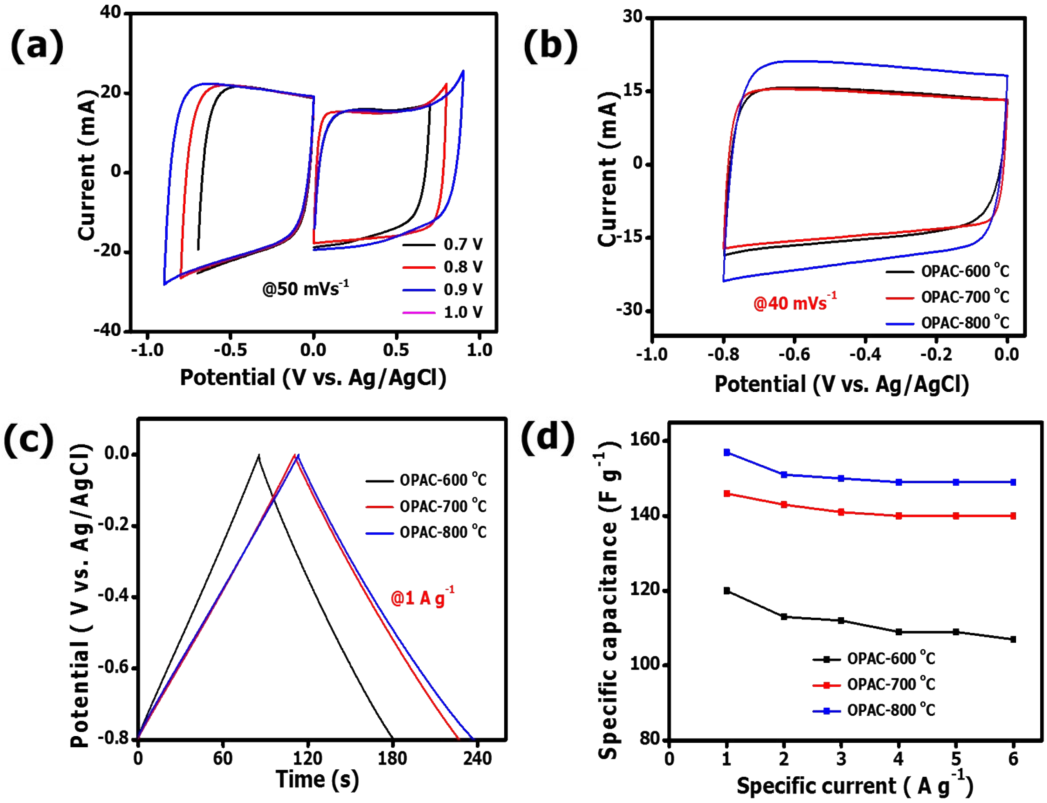

Figure S3: Onion-derived activated carbons: (a) CV curves at a scan rate of 50 mV s^{-1} at varying operating potential window, (b) CV curves at a scan rate of 40 mV s^{-1} at an operating potential of -0.8 V to 0.0 V, (c) respective galvanostatic charge-discharge plots and (d) specific capacitance calculated versus specific current for the negative potential windows.
